# Tissue Modeling and Analyzing with Finite Element Method: A Review for Cranium Brain Imaging

**DOI:** 10.1155/2013/781603

**Published:** 2013-02-05

**Authors:** Xianfang Yue, Li Wang, Ruonan Wang

**Affiliations:** Mechanical Engineering School, University of Science and Technology Beijing, Beijing 100083, China

## Abstract

For the structure mechanics of human body, it is almost impossible to conduct mechanical experiments. Then the finite element model to simulate mechanical experiments has become an effective tool. By introducing several common methods for constructing a 3D model of cranial cavity, this paper carries out systematically the research on the influence law of cranial cavity deformation. By introducing the new concepts and theory to develop the 3D cranial cavity model with the finite-element method, the cranial cavity deformation process with the changing ICP can be made the proper description and reasonable explanation. It can provide reference for getting cranium biomechanical model quickly and efficiently and lay the foundation for further biomechanical experiments and clinical applications.

## 1. Background

There have been many methods used to study the biomechanics of tissue structure today. Biological tissues are widely used in animal experiments, physical experiments, and *in vitro *(cadaver) experiments, all of which are included in human *in vitro *experiments. Animal experiments can provide the physiopathological responses in living bodies, but animals *in vivo* cannot be used to solve all the problems regarding human characteristics, because their tissue structure and functions are different from those of humans. Physical experiments only offer limited effects for lacking of geometrical structure characteristics of living bodies. Human cadaver model experiments would yield experimental data closest to practical outcomes because of the similar geometrical structure characteristics of human cadavers with the living bodies, but the cost is high and simulation of the characteristic changes of living body is difficult when testing.

At present, most biomechanical methods can only detect the external mechanical changes of human specimens. Thus, it is difficult to fully reveal the mechanisms of interaction of each part, and the internal structure displacement and internal stress change can be only presumed according to the pathological process, which lacked objective experimental support. Three-dimensional image reconstruction and finite element simulation can solve these problems. Computer simulation experiments, such as finite element models, can reflect the situations after reconstructing repeatedly simulating experiments or changing some parameters according to the biomechanical characteristics, which cannot be obtained by other methods. Finite element method (FEM) has been shown to be an effective analysis of theoretical biomechanics. It uses the mathematical model of mechanics to perform numerical value mechanical analysis and reduce mathematical behavior characteristics of engineering system. Mathematical characteristics of tissue simulated in the form of mathematics include node, element, material attribute, loading, and boundary condition, which are acquired based on physical prototype. An alteration of local structure, parameters, and loading can simulate the displacement and stress in any place and explain the stress change of tissue during physiopathological process to acquire overall information.

## 2. Introduction

The creation of digital copies of humans is an innovation in anatomy education. These 3D meshes create a new alternative for students and researchers. It also allows the remote access and the manipulation of the pieces without manual consuming. The 3D visualization of the human skull represents an advance in the interpretation of images, because it makes possible the volumetric analysis of the anatomical structures and it evidences more clarity in its space configurations and its relations with other organs [1]. Indeed, research is focusing more and more on the acquisition of models and simulation parameters from real people rather than procedurally simulating their appearance and movements [2].

The “Monro [3]-Kellie [4] doctrine” states that an adult cranial compartment is incompressible, and the volume inside the cranium is a fixed volume thus creating a state of volume equilibrium, such that any increase of the volumes of one component (i.e., blood, CSF, or brain tissue) must be compensated by a decrease in the volume of another. If this cannot be achieved, then pressure will rise and once the compliance of the intracranial space is exhausted then small changes in volume can lead to potentially lethal increases in ICP. The compensatory mechanism for intracranial space occupation obviously has limits. When the amount of CSF and venous blood that can be extruded from the skull has been exhausted, the ICP becomes unstable and waves of pressure develop [5]. As the process of space occupation continues, the ICP can rise to very high levels and the brain can become displaced from its normal position. Dr. Sutherland [6] firstly perceived a subtle palpable movement within the bones of cranium. Dr. Upledger [7] discovered that the inherent rhythmic motion of cranial bones was caused by the fluctuation of CSF. Accordingly, the cranium can move and be deformed as the ICP fluctuates.

Clinical data shows that increased pressure within the brain matter caused by lesions or swelling within the brain matter itself resulted in raised intracranial pressure (ICP) is a serious and often fatal condition, and compression of vital brain structures and blood vessels can lead to serious, permanent neurologic deficits or even death. It is hard to perform biomechanical analysis of stress and strain of cranial cavity with the changing ICP, even through experiments. Therefore, a mechanical model was established through the use of three-dimensional finite element method to provide an effective pathway for biomechanical research of cranium brain.

## 3. Finite Element Methods

### 3.1. Finite Element Method (FEM)

Finite Element Method (FEM) is an integrated product of many disciplines, including mechanics, mathematical physics, computational methods, and computer technology. The three research methods, theoretical analysis, scientific experiments, and scientific computing, have been applied to study the nature problems. Because of the limitations of scientific theory and experiments, the scientific computing becomes one of the most important research tools. As one of ways to carry out the scientific computing, the finite element method can almost analyze any complex engineering structure so as to obtain various mechanical properties in most engineering fields.

The essence of the finite element method is that a complex continuum is divided into a limited number of simple elements transformed the infinite freedom degree to the limited one, and the differential equation to the algebraic equations of finite parameters. While analyzing the problems of engineering structure with the finite element method, after the discretization of an ideal discrete body, it is one of mainly discussed contents of the finite element theory of how to ensure the convergence and stability of numerical solution. The convergence of numerical solution is related to the element division and shape. During the solving process, as the basic variable, the displacement is usually solved through the virtual displacement or minimum potential energy principle. 

### 3.2. The Basic Steps of the Finite Element Method

**Figure 1:**
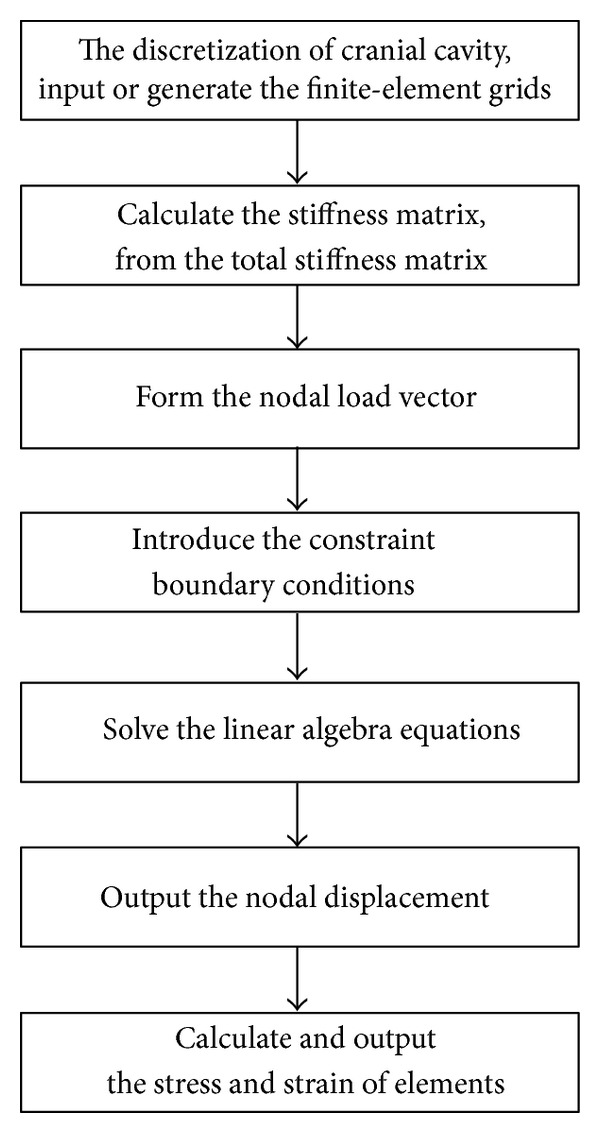
Block diagram of numerical solution steps of cranial cavity with the finite element method.

The solving steps for the strains of cranial cavity with the ICP changes are shown in Figure 1. The specific numerical solution process is [8]

1. identifying the discretized cranial cavity,
2. selecting the displacement mode,
3. analyzing the mechanical properties of elements and deriving the element stiffness matrix,
4. collecting all relationship between force and displacement and establishing the relationship between force and displacement of cranial cavity,
5. solving the nodal displacement,
6. classifying the nodal displacement and the strain and stress in each element then calculating.

### 3.3. The Application of Finite Element Method in Biomechanics

It is a great achievement for the finite element method to be applied in the quantitative research in the life science. In particular, its great superiority is demonstrated in the research on human biomechanics. After a long evolution of human labor, the human skeleton has almost formed a perfect mechanical structure. However, the mechanics experiment is almost impossible to directly develop while the mechanical structure of human body is studied. At that time, it is an effective means that the finite element numerically simulates the mechanical experiments.

In 1960s, the finite element method was initially applied in the cardiovascular system study on mechanical problems. From the 1970s, the orthopedic biomechanics research began to be initially applied to spine. After 80 years, the applicable range is gradually extended to craniofacial bone, mandible bone, femur [9], teeth [10], joint [11, 12], cervical [13], lumbar [14], and its subsidiary structure in the bio-mechanics fields as illustrated in the following points.

1. Improvement and optimization design of equipment.
2. The mechanical simulation experiment by finite element model.
3. Nowadays, the finite element method has been widely used in the country and has made a lot of successes. Particularly, there is more certain guidance in the clinical application. In biomechanics, the finite element application, there are a large number of opportunities to research the shape and structure of finite element model.

## 4. Finite Element Modelling of the Viscoelastic ****Human Cranial Cavity

### 4.1. The Simple Finite Element Modelling of the Human Skull-Dura Mater System

**Figure 2:**
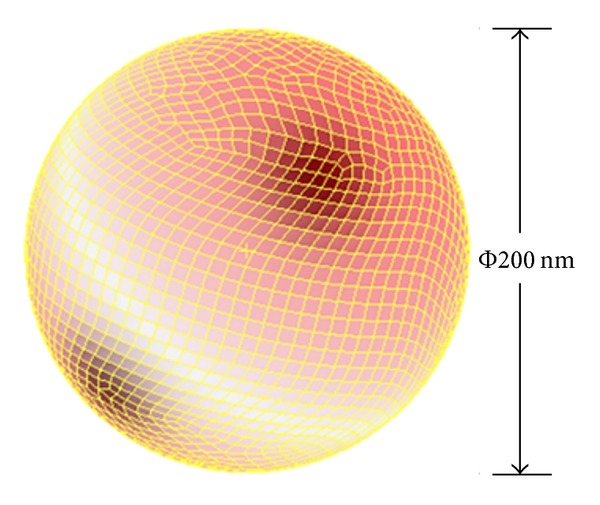
The sketch of 3D cranial cavity and grid division.

The craniospinal cavity may be considered as a balloon. For the purpose of our analysis, we adopted the model of hollow sphere (Figure 2). We presented the development and validation of a 3D finite element model intended to better understand the deformation mechanisms of human skull corresponding to the ICP change. Based on the established knowledge that cranial cavity is importantly composed of skull and dura mater, a thin-walled structure was simulated by the composite shell elements of the finite element software [15].

Of course, the structure, dimension, and characteristic parameter of human skull must be given before the calculation. The thickness of calvaria [16] varies with the position, age, gender, and individual, so does dura mater [17]. Tabula externa and interna are all compact bones and the thickness of Tabula externa is more than that of Tabula interna. Diploe is the cancellous bone between Tabula externa and Tabula interna [18]. The parietal bone is the transversely isotropic material, namely, it has the mechanical property of rotational symmetry in the axially vertical planes of skull [19]. The important mechanical characteristic of cancellous bone is viscoelasticity, which is generally considered as the semiclosed honeycomb structure composed of bone trabecula reticulation. The main composition of cerebral dura mater, a thick and tough bilayer membrane is the collagenous fiber, which has the characteristic of linear viscoelasticity [20]. And the thickness of dura mater obviously varies with the changing ICP [21]. The mechanical performance of skull is isotropic along the tangential direction on the surface of skull bone [22], in which the performance of compact bone in the Tabula externa is basically the same as that in the Tabula interna [23]. Thus both cancellous bone and dura mater can be regarded as isotropic materials. And the elastic modulus of fresh dura mater varies with the delay time [24].

ICP is not a static state, but one that influenced by several factors. But so far there are almost no records of the actual human being's ICP in clinic. The geometry and structure of monkey's skull, mandible, and cervical muscle are closer to those of human beings than other animals. So the ICP of monkeys [25] can be taken as the reference to that of human beings. The brain appears to be mild injury when ICP variation is about 2.5 kPa, moderate injury when ICP variation is about 3.5 kPa, and severe injury when ICP variation is about or more than 5 kPa. Therefore, we carried out the following theoretical analysis with the ICP scope from 1.5 kPa to 5 kPa.

**Figure 3:**
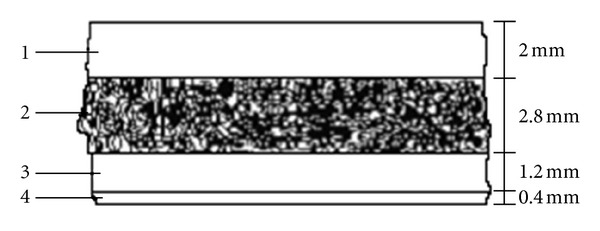
Sketch of layered sphere. The thin-walled structure of cranial cavity is mainly composed of Tabula externa, Diploe, Tabula interna, and dura mater.

In this paper, the finite element software MSC_PATRAN/NASTRAN, Ansys, and Mimics are applied to theoretically analyze the deformation of human skull with the changing ICP. The skull is a layered sphere constructed in a specially designed form with a Tabula externa, Tabula interna, and a porous Diploe sandwiched in between (Figure 3). The external diameter of cranial cavity is about 200 mm. The thickness of shell is the mean thickness of calvarias. The average thickness of adult's calvaria is 6.0 mm, that of Tabula externa is 2.0 mm, Diploe is 2.8 mm, Tabula interna is 1.2 mm, and dura mater in the parietal position is 0.4 mm.

Considering the characteristic of compact bone, cancellous bone, and dura mater, their mean elastic modulus and Poisson ratios are 1.5 × 10^4^ MPa, 4.5 × 10^3^ MPa [26], 1.3 × 10^2^ MPa [27], and 0.21, 0.01, 0.23, respectively.

**Figure 4:**
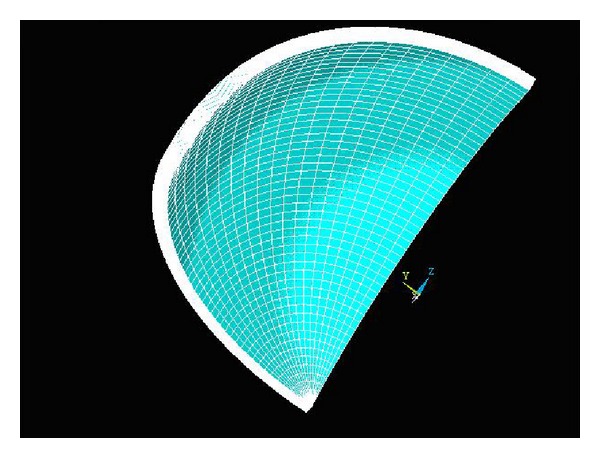
Finite element model of 1/8 cranial cavity shell.

In addition, human skull has the viscoelastic material [28]. Considering the viscoelasticity of human skull and dura mater, we use the viscoelastic option of the Ansys finite element program to analyze the strains on the exterior surface of human skull as ICP changing. According to the symmetry of 3D model of human skull, the preprocessor of the Ansys finite element program is used to construct an 1/8 finite element model of human skull and dura mater consisting of 25224 nodes and 24150 three-dimensional 8-node isoparametric solid elements, shown in Figure 4.

In the finite element software Ansys, there are three kinds of models to describe the viscoelasticity of actual materials, in which the Maxwell model is the general designation for the combined Kelvin and Maxwell models. Considering the mechanical properties of human skull and dura mater, we adopt the finite element Maxwell model to simulate the viscoelasticity of human skull-dura mater system. The viscoelastic parameters of human skull and dura mater are, respectively, listed in Tables 1 and 2.

**Figure 5:**
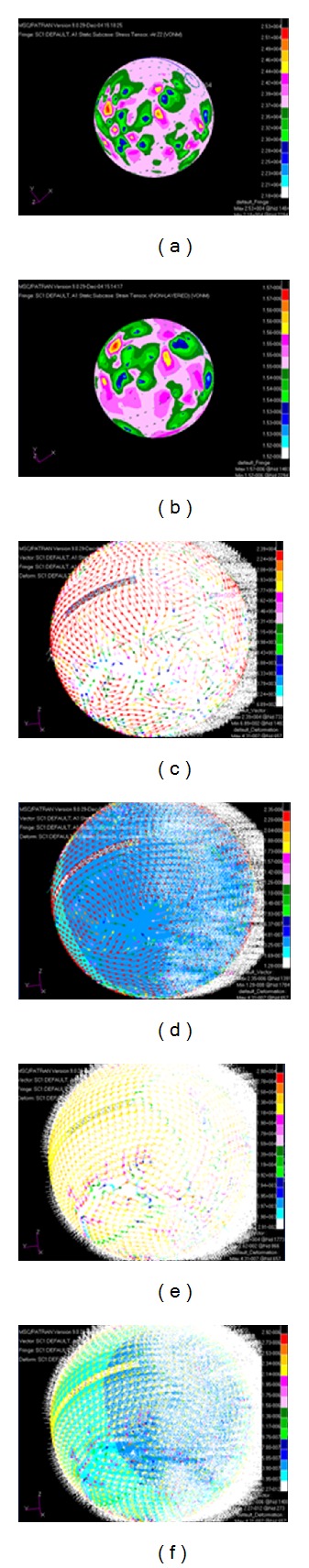
The stress and strain distribution ignoring viscoelasticity of human skull and dura mater. (a) Stress distribution, (b) strain distribution, (c) the maximal stress vector distribution, (d) the maximal strain vector distribution, (e) stress vector distribution, and (f) strain vector distribution.

After ignoring the viscoelasticity of human skull and dura mater, the stress and strain graphs of skull bone are shown in Figure 5 when ICP variation is raised up to 2.5 kPa.

**Figure 6:**
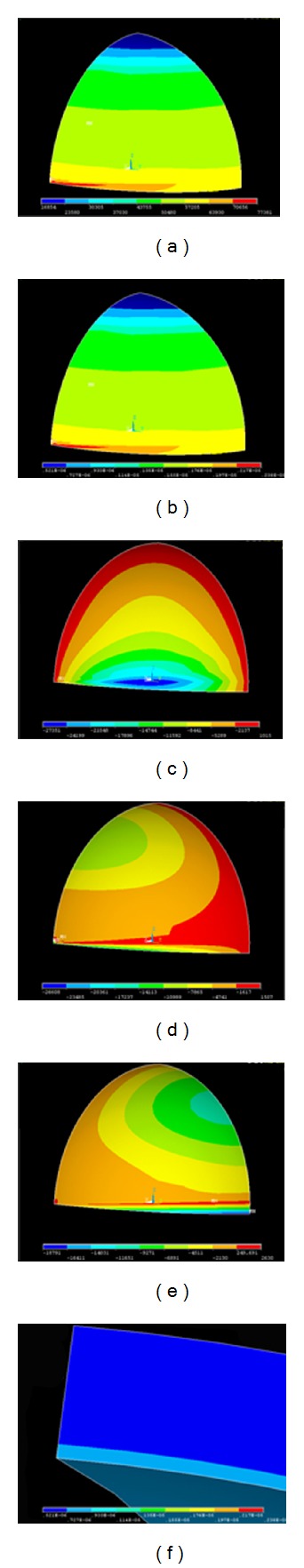
The stress and strain distribution considering viscoelasticity of human skull and dura mater. (a) Stress nephogram, (b) strain nephogram, (c) *XY* shear stress nephogram, (d) *XZ* shear stress nephogram, (e) *YZ* shear stress nephogram, and (f) interbedded strain change nephogram in the human dura mater and skull.

Figure 6 is the analytic graphs of stress and strain by considering the viscoelasticity of human skull and dura mater with finite element software Ansys when ICP variation is raised up to 2.5 kPa.

**Figure 7:**
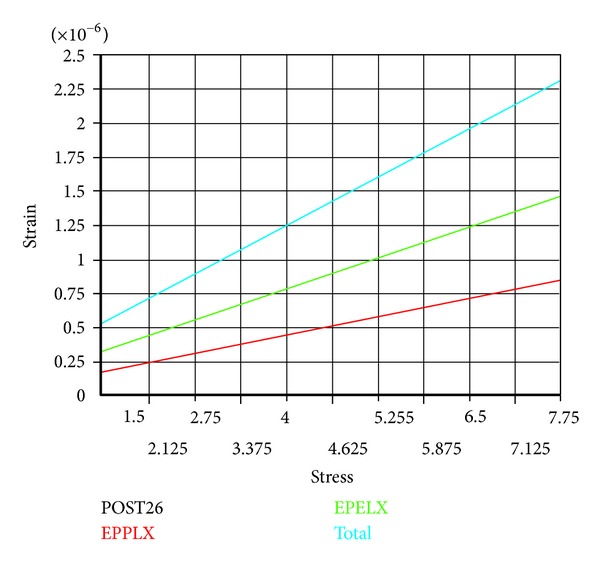
Curves among total, elastic, and viscous strain when the ICP increment is 2.5 kPa. Here EPELX is elastic strain curve, and EPPLX is viscous strain curve. The viscous strain is about 40% of total strain.

From the relationships between total, elastic, and viscous strains of human skull and dura mater in Figure 7, the viscous strains account for about 40% and the elastic strains are about 60% of total strains with the increasing ICP.

**Figure 8:**
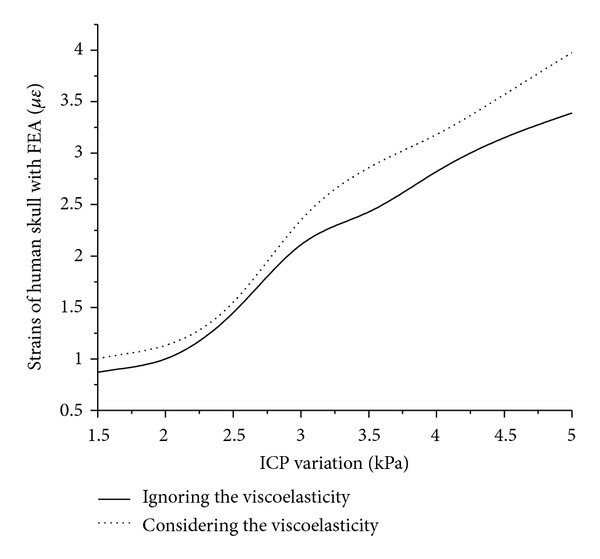
The strain curves of finite element simulation under the conditions of ignoring and considering the viscoelasticity of human skull and dura mater with the changing ICP from 1.5 kPa to 5 kPa.

After considering and ignoring the viscoelasticity of human skull and dura mater, the stresses and strains of cranial cavity are shown in Figure 8 as the ICP changing from 1.5 kPa to 5 kPa with the finite element software. The deformation scope of human skull is theoretically from 0.9 *με* to 3.4 *με* as the ICP changing from 1.5 kPa to 5.0 kPa. It shows that the stress and strain distributions on the exterior surface of human skull are well proportioned and that the stress and strain variation on the exterior surface of cranial cavity is relatively small corresponding to the ICP change.

### 4.2. Approximate Symmetry of Cranial Cavity with Therapeutic Hypothermia

#### 4.2.1. The Geometric Model

Reconstructions of the finite element model of cranial cavity are mostly through the multi-CT scanning technology at home and abroad [29, 30], which is simple and of high precision. However, the object being scanned is only a single individual. It is difficult to scan multiple images of individual unity for the universal data. Based on the average measured data of human skull of 104 Chinese people from 18 to 76 years of age, including 67 male and 37 female [31], the three-dimensional model of cranial cavity is directly drawn in the Ansys program.

**Figure 9:**
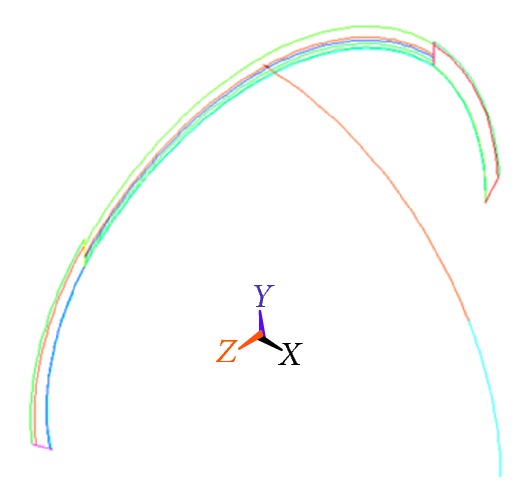
Skeleton of the cranial cavity trendline.

This paper studied importantly the deformation of cranial cavity, including brain tissue, cerebrospinal fluid, and brain blood flow with the ICP changes. So the model of cranial cavity was properly simplified: only the cavity in which the brain lies, that is, a closed cavity, is made up of the parietal bone, occipital, frontal, and temporal bones, and a layer of dura mater. For the approximate symmetry of person's head, the 1/2 cranial cavity model is built in this paper (Figure 9).

**Figure 10:**
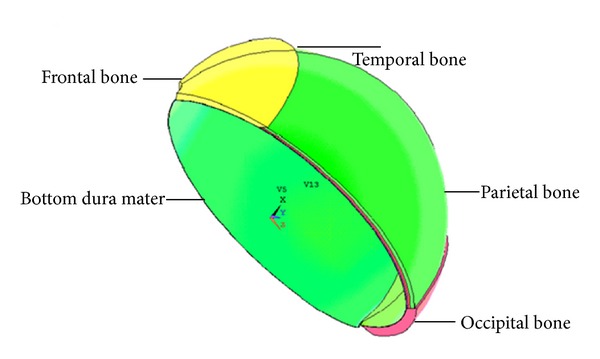
3D model of 1/2 cranial cavity.

**Figure 11:**
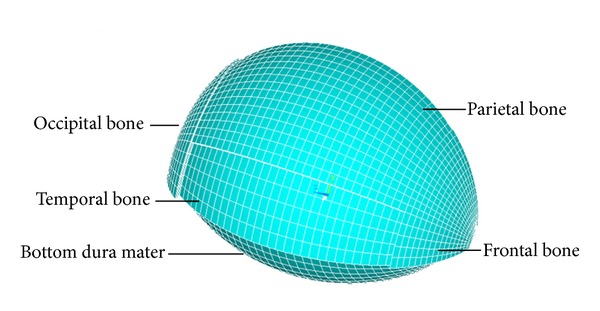
Finite element meshes of 1/2 cranial cavity.

In Figure 9, the thickness of parietal, frontal, occipital, and temporal bones, as well as dura mater, is, respectively, 5.3560 mm, 6.5558 mm, 7.5286 mm, 2 mm, and 0.4 mm [32, 33]. From outside to inside each layer in turn, the thickness of external compact bone and the Diploe and internal compact bones is about 3 mm, 1.8 mm, and 1 mm. Due to the smoothness, the parietal bone is regarded as the main measured position. By the Extrude command, in Figure 10 the volume can be formed. After bonding the border among the frontal, parietal, occipital, and temporal bones and the dura mater with the glue command, the model of cranial cavity is shown in Figure 11.

#### 4.2.2. The Meshing and Load

The three-dimensional finite element model of cranial cavity is considered as a composite structure composed of the skull and dura mater. The hexahedral grid was adopted to mesh the entire cranial cavity. The adjacent parts were dealt with by the Glue command, and the grid refinement with the Meshing-Modify Mesh command was used as the irregular mesh to the edge, sharp, or irregular position. Thus the 1/2 finite element model of the cranial cavity, including the parietal, prefrontal, occipital, and temporal bones and dura mater, and the cell type is block unit. Figure 11 is the meshing diagram of 1/2 cranial cavity, in which there are 9,700 hexahedral element and 27,256 nodes.

The mild hypothermia is the main temperature environment for the treatment of brain injury, intracranial hypertension, and so on. Mild hypothermia treatment of severe traumatic brain injury in recent years is another important means [34]. So the optimal temperature of treatment is consistent with the scope among 32°C~35°C at home and abroad [35]. Thus the temperature load of 33.5°C is exerted to the outer surface of human skull to simulate the mild hypothermia therapy.

The finite element method extensively solves the biomechanical problem in the medical fields. Compared to other biomechanical modellings, the finite element method can more accurately express the human body geometry and architecture. Therefore, the Ansys finite element software is in this paper used to reconstruct the three-dimensional cranial cavity of human being with the mild hypothermia treatment.

**Figure 12:**
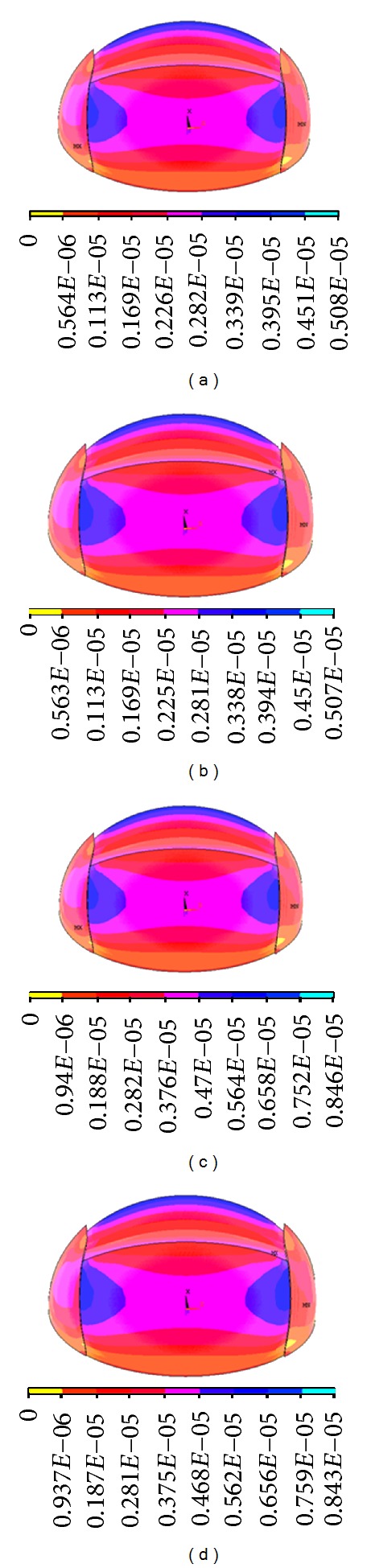
Strain graph. (a) Without hypothermia treatment when ICP is 3.0 kPa, (b) with hypothermia treatment when the ICP is 3.0 kPa, (c) without hypothermia treatment when the ICP is 5.0 kPa, and (d) with hypothermia treatment when the ICP is 5.0 kPa.

Figure 12 is the strain diagrams of cranial cavity under the mild hypothermia environment and normal temperature conditions while the ICP is 3.0 kPa. It shows that the strains decreased about 0.001 *με* under the mild hypothermia environment than those under the normal temperature conditions during the same circumstance of ICP changes.

## 5. 3D Finite Element Cranial Cavity Model of ****Human Beings

### 5.1. Materials and Methods

#### 5.1.1. CT Scan

A healthy male volunteer, aged 40 years old, with body height 176 cm, weighing 75 kg, was included in this study. The volunteer explained no history of cranium brain. Common projections (posterior-anterior, lateral, dual oblique, hyperextension, and hyperflexion) were made to exclude cranium brain degenerative disorders, cranial instability, and brain destruction. 

**Figure 13:**
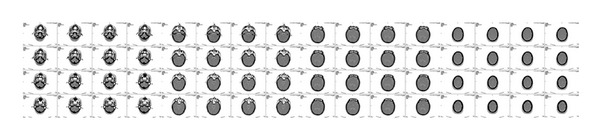
CT scan image of cranial cavity.

Spiral CT scans (1 mm thickness) were output in the JPG image file format and saved in the computer. Prior to experiment, informed consent was obtained from this volunteer, shown in Figure 13.

#### 5.1.2. Experimental Equipment

High-performance computer (Lenovo, X200) and mobile storage equipment were used. Solid modeling software Mimics 13.0 (Materiaise's interactive medical image control system) was used in this study. As a top software in computer-aided design, Mimics 13.0 provides many methods of precise modeling and has been widely used for precise processing. Its equipped Ansys and Partron finite element analysis module sequence were used for finite element analysis, and then the strain and deformation regularity of the real human cranial cavity were simulated with the changing ICP.

#### 5.1.3. Flowchart of AutoCAD

**Figure 14:**
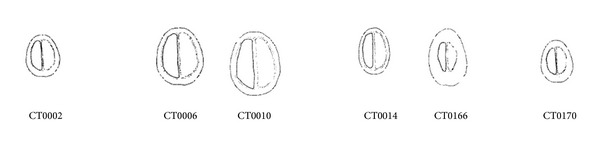
AUTO CAD simulative spline curve.

**Figure 15:**
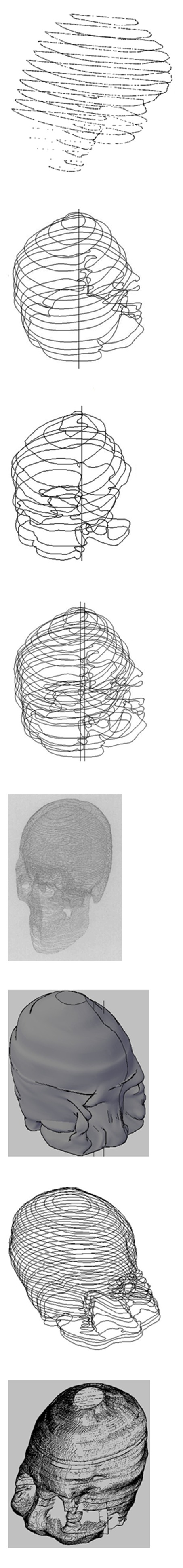
3D space model of cranial cavity with AutoCAD.

By the insert function of AutoCAD, the spline curves of CT pictures will be simulated, shown in Figure 14. And the 3D space model is made as in Figure 15.

It can be seen that the 3D model of cranial cavity with AutoCAD software failed and cannot meet the requirements of analysis because the “poor set” or “smoke shell” commands cannot be executed.

#### 5.1.4. Flowchart of Finite Element Method

*Methods*

*(i) Image Boundary Tracking. *A self-programmed image boundary automatic recorder was used to acquire cranial information along the superior border to inferior border of cranium brain. The spatial boundary was recorded layer by layer. Following point selection from interior and exterior border of images, two-dimensional space coordinates were automatically recorded and saved in the  .CDB form. After conversion, this file can be directly input into mimics or CAD software. 

*(ii) Location. *A coordinate system was determined. The CT scanning image starting from the lowest layer of cranium brain was set as the working background. CT scan was performed based on a fixed coordinate axis, with a known layer interval and magnification proportion. The spatial three-dimensional coordinate of each point in the image could be determined through drawing the horizontal coordinate of each point and referencing scanning interval. When CT machines recorded each layer of images, all images were in the same scanning range, which equaled to cranial location of two-dimensional CT images from each layer in the scanning direction. Calibration of two-dimensional images could be performed if the scale bar of CT scan images were given. In addition, each layer of image was scanned with some interval in the longitudinal direction, which was equivalent to calibration in the third-dimensional direction. 

**Figure 16:**
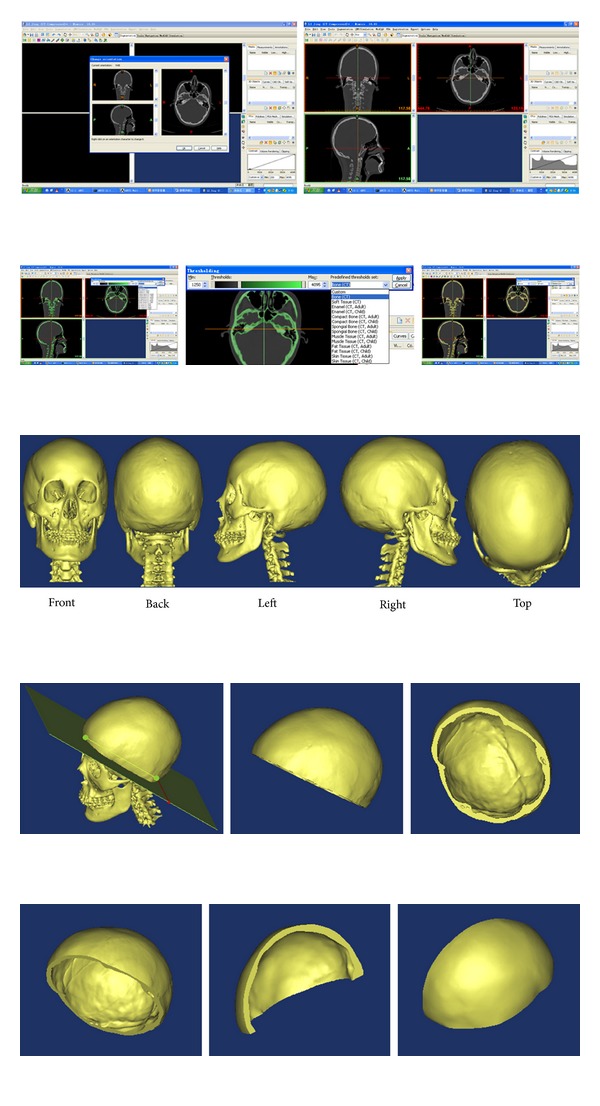
3D model of cranial cavity.

*(iii) Image Reconstruction. *In accordance with the sequence of CT scans and according to the scale bar and scan interval of CT-faulted image, geometry data of each layer were input into the preprocessing module of finite element software to establish a geometry model in the rectangular coordinate in the sequence of point, line, area, and solid. The transverse plane of CT scan was parallel to *xy* plane, and the longitudinal plane was along the *z*-axis. Three-dimensional reconstruction process is shown in Figure 16.

**Figure 17:**
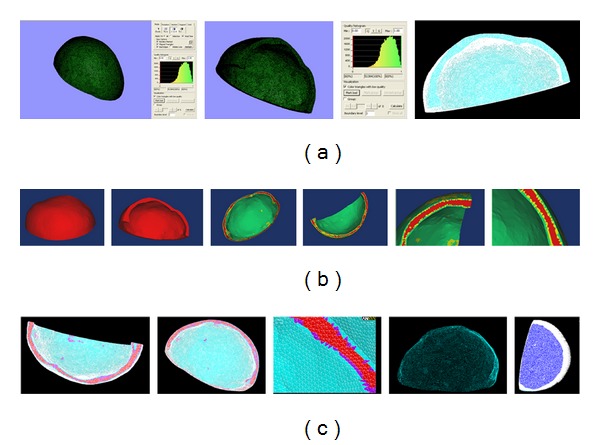
The cranial cavity model after smooth processing and mesh division.

Since the cranial cavity model is for smooth processing and mesh division in the Mimics and Ansys software, the figures are shown in Figure 17.

The property of viscoelastic materials is adopted in the Prony Model. The shear modulus and volume modulus are, separately,

(1) $$\begin{array}{c} G\left(t\right)=G_{\infty }+\sum_{i=1}^{n_{G}}G_{i}\exp\left(-\frac{t}{\tau_{i}^{G}}\right),\\ K\left(t\right)=K_{\infty }+\sum_{i=1}^{n_{K}}K_{i}\exp\left(-\frac{t}{\tau_{i}^{K}}\right), \end{array}$$

where *G*_*∞*_ and *G*_*i*_ are shear modulus, *K*_*∞*_ and *K*_*i*_ are volume modulus, and *τ*_*i*_^*G*^ and *τ*_*i*_^*K*^ are the slack time of series Prony components.

### 5.2. Results

#### 5.2.1. Reconstruction Results

**Figure 18:**
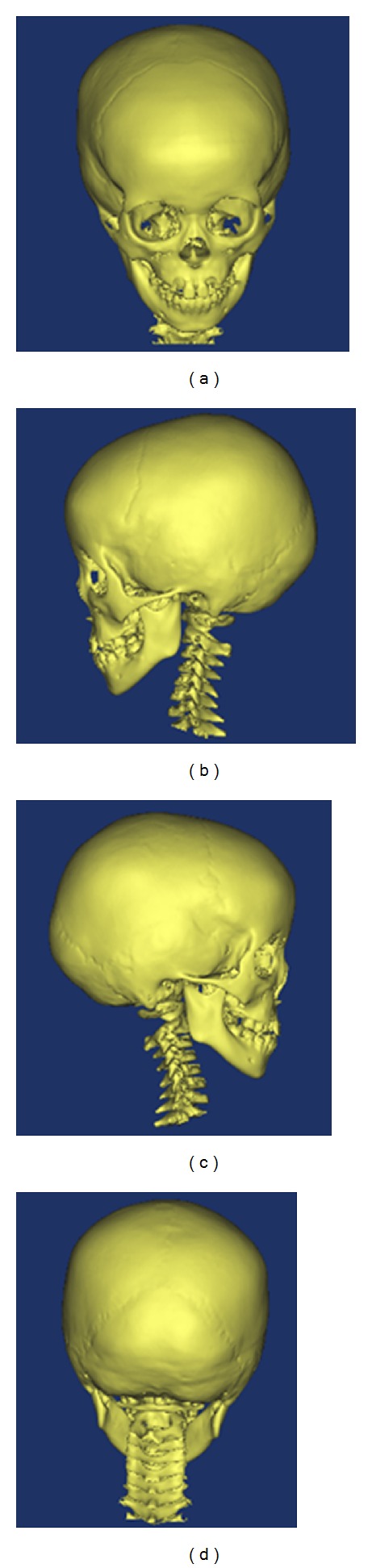
Each view drawing on the 3D finite element model in of cranial cavity.

The fitted curves were assigned into different layers to construct the solid structure of bone (Tabula externa, Tabula interna, Diploe sandwiched in between, dura mater), and spongy. During reconstructing the structure of Tabula externa, Tabula interna, Diploe sandwiched in between, and dura mater, each part was established independently, and then the whole part was set up. The three-dimensional finite element models in each direction of cranial cavity are shown in Figure 18.

#### 5.2.2. Model Validation

**Figure 19:**
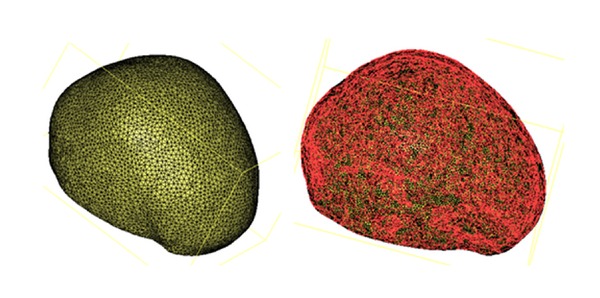
3D finite element model of cranial cavity is meshed.

**Figure 20:**
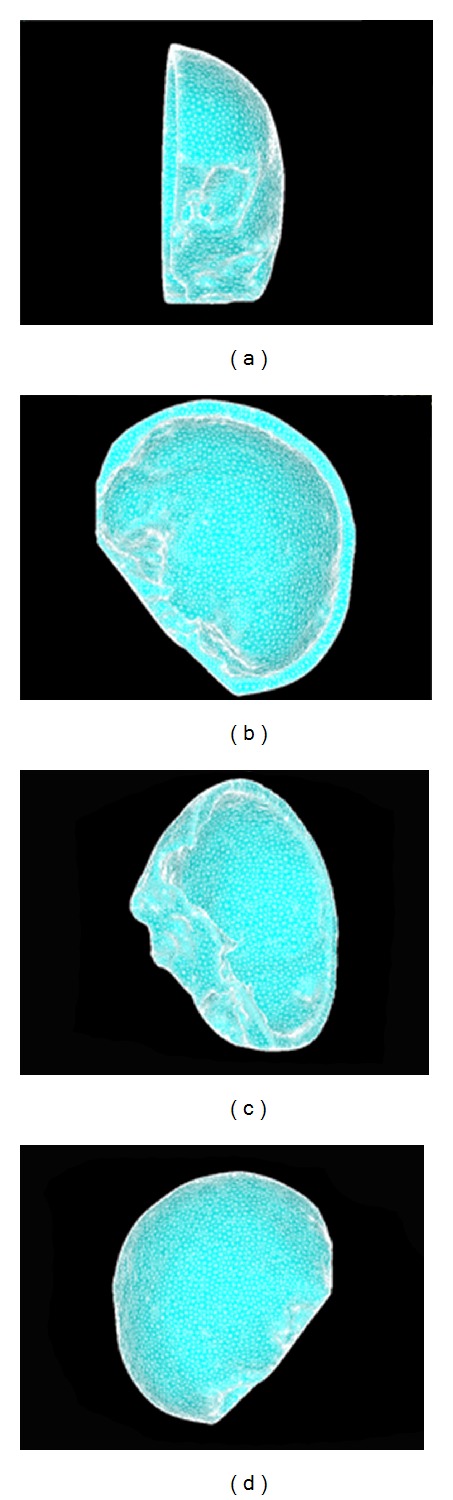
Finite element model of 1/2 cranial cavity.

**Figure 21:**
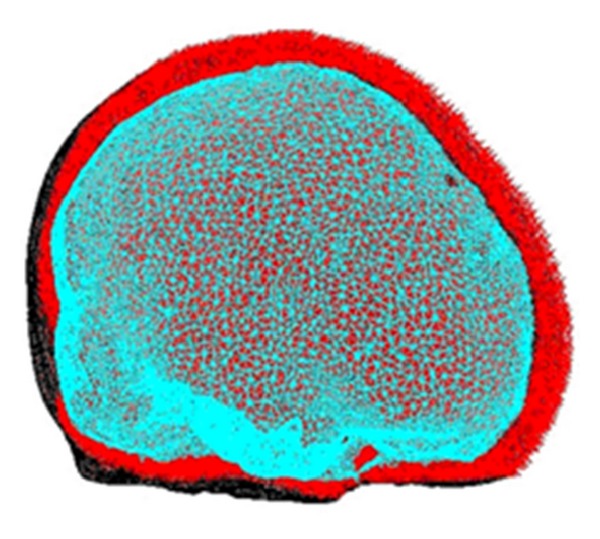
Loads on the finite element model of 1/2 cranial cavity.

**Figure 22:**
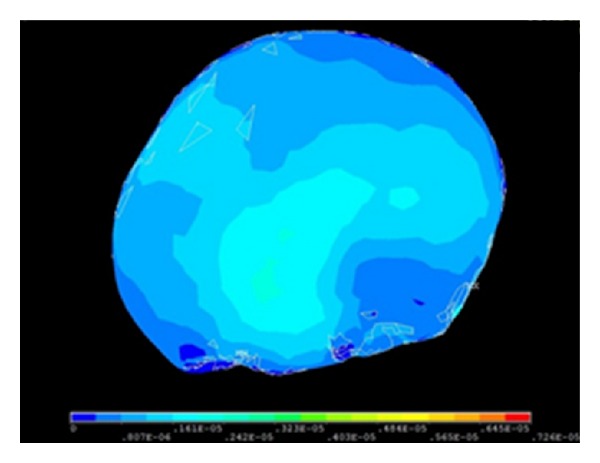
Strain graph when the ICP is 3.0 kPa.

**Figure 23:**
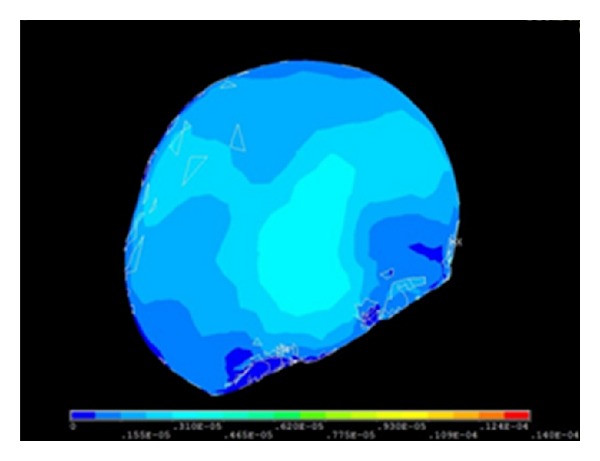
Strain graph when the ICP is 5.0 kPa.

**Figure 24:**
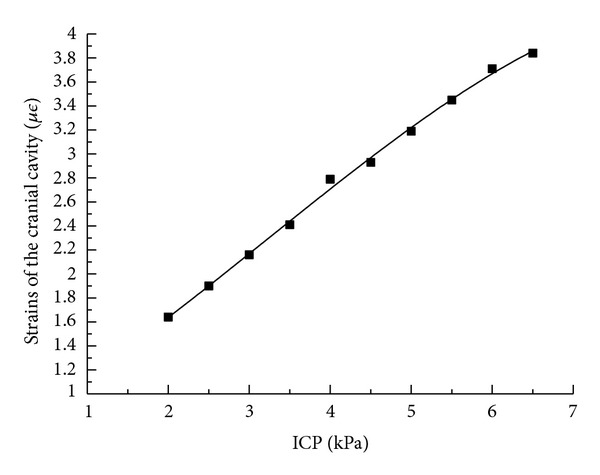
Strains curve of cranial cavity with the ICP variation.

**Figure 25:**
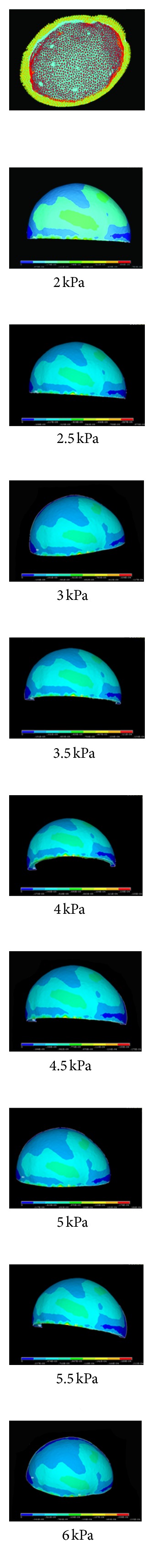
Analysis of cranial cavity with Prony model.

Following type selection, finite element mesh generation was performed in the above-mentioned models which were given material characteristics. Then, through simulating practical situation, boundary condition was exerted as well as the proper numerical process. And the three-dimensional analysis was performed. 3D finite element model of cranial cavity is meshed in Figure 19. Simulation analysis of cranium brain three-dimensional finite element model is shown in Figures 20, 21, 22, 23, 24, and 25.

#### 5.2.3. Analysis of Cranial Cavity with Prony Model

The analysis results of cranial cavity with material properties of Prony model in the Mimics and Ansys software are shown in Figure 25.

**Figure 26:**
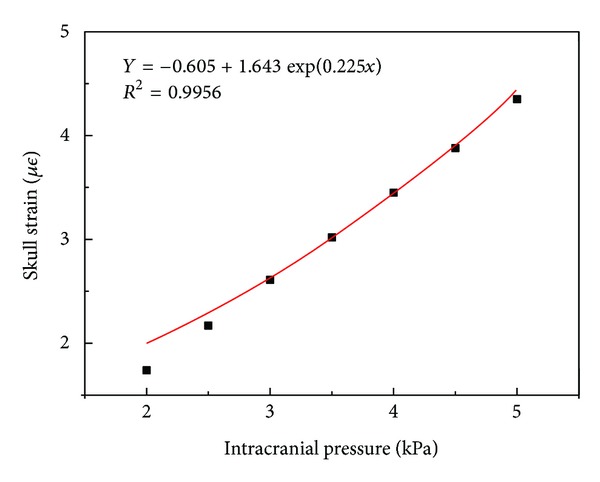
The relationship of skull strain and intracranial pressure.

After fitting the relationship of skull strain and intracranial pressure, the curve is shown in Figure 26.

It can be seen that the strains of cranial cavity are close to the index growth with the increasing intracranial pressure. The relationship of skull strain and intracranial pressure is *y* = −0.605 + 1.643exp(0.225*x*) and the confidence is 0.9956.

## 6. Conclusion and Discussion

### 6.1. Conclusion

Biomechanical model has been shown to play a key role in study of cranium brain, because it can be used to investigate the pathogenesis through model observation, thereby to propose the strategy of diagnosis and treatment.

Owing to irregular geometry and nonuniform composition of cervical spine cranium bone as well as impossible human mechanical tests, increasing attention has been recently paid to finite element method included in the biological study of cranium brain injury because this method exhibits unique advantages in analysis of complex structure.

Experimental results are the best method to verify model accuracy. When exerting persistent pressure to vertebral spine, nonlinear computation is supplemented to the two-dimensional unit calculation of ligament structure, which corresponds more to human mechanical structure. Statics solver exhibits the self-testing function and can automatically analyze computation process, report errors, and control error range. The displacement graph, stress graph, and isogram drawn by postprocessor visualize the distribution ranges of stress or strain loaded on each part of cranium brain with the changing ICP. When loads are vertically added, the stress on the posterior wall of cranium brain, as well as on the end plate and the posterior part of intervertebral discs, relatively centralizes. 

### 6.2. Discussion

Finite element analysis is an important mean to simulate human structural mechanical function in the field of biomechanics. A human finite element model with physical material characteristics under proper simulated *in vivo* condition can be used to effectively analyze the physical characteristics of human structure, for example, stress/strain of structure, modal analysis, exterior impact response, and fatigue test. With further understanding of cranium brain diseases, some complex models have not been developed, for example, finite element models of head and cranial cavity used to study the physiopathological influences of cervical spine loading in some complex exercises on cranium brain and soft tissue. Finite element analysis exhibits unexampled advantages in the biomechanical study of a severe medical brain problem. Theoretically, finite element method can simulate nearly all biomechanical experiments. Moreover, this method can better describe the interior changes of living body than practical study. Finite element method, as an emerging technique, has a broad developing space. However, it is a theoretical simulation analysis, and only in conjunction with clinical detection and observation can it truly reflect the occurrence and progression of cranium brain disease and provide evidence for predicting curative effects, thereby exhibiting a synergic effect with clinical outcomes. The present model is only a initial research. It cannot reflect the changes between individual interior parts and between individuals in terms of bone contour and material characteristics. The present model is only a cranium brain motion segment. Its simulation analysis results might differ from the results from multiple motion segments. Actually, when much difference and many uncertainties exist between individuals, model simplification and idealization is to strengthen some research aspects, which removes experimental inherent difference. Of course, establishment of a finite element mechanical model is to provide mechanical methods for clinical and experimental studies. The present model needs further improvements due to some limitations, that is, unable to reflect some complex condition, but it ensures the geometry data and material characteristic approximation for application of multiple tools equipped by various softwares during the process of model establishment. In addition, finite element method, as one of tools used in the biomechanical field, can qualitatively analyze the stress change of cranium brain interior parts when bearing forces. Only by changing local structure or materials can the present model established by finite element method simulate the common clinical situation and the effects of intervention on ICP force. The present model should be further improved in the clinical and experimental processes.

## Figures and Tables

**Table 1:** Coefficients for the viscoelastic properties for human skull.

	Elastic Modulus (GPa)		Viscosity (GPa/s)	Delay time *τ* (s)	
	*E*_0_	*E*_1_	η	τ_γ_*	τ_d_*
Compression	5.69 ± 0.26	42.24 ± 2.09	26.9 ± 1.5	2022 ± 198	2292 ± 246
Tension	13.64 ± 0.59	51.45 ± 2.54	57.25 ± 4.27	3180 ± 300	4026 ± 372

*τ_γ_ = η/*E*_0_ + *E*_1_, τ_*d*_ = η/*E*_1_.

**Table 2:** Creep coefficients for the viscoelastic properties for fresh human dura mater.

	Elastic Modulus (MPa)				Delay time *τ* (s)		
	*E*_0_	*E*_1_	*E*_2_	*E*_3_	τ_1_	τ_2_	τ_3_
Dura mater	16.67	125.0	150.0	93.75	40	10^4^	10^6^
